# Patient perspectives on priorities for research on conventional and sex- and gender-related cardiovascular risk factors

**DOI:** 10.1007/s12471-020-01497-9

**Published:** 2020-10-06

**Authors:** R. Bolijn, I. Schalkers, H. L. Tan, A. E. Kunst, I. G. M. van Valkengoed

**Affiliations:** 1 Department of Public and Occupational Health, Amsterdam Public Health research institute, Amsterdam University Medical Center, AMC/University of Amsterdam, Amsterdam, The Netherlands; 2 Harteraad, Den Haag, The Netherlands; 3 Department of Cardiology, Amsterdam Cardiovascular Sciences, Amsterdam University Medical Center, AMC/University of Amsterdam, Amsterdam, The Netherlands; 4 Netherlands Heart Institute, Utrecht, The Netherlands

**Keywords:** Sex differences, Gender differences, Cardiovascular risk factors, Prevention, Patient participation, Research priorities

## Abstract

**Background:**

Recently, cardiovascular disease (CVD) research has focused on sex- and gender-related cardiovascular risk factors, in addition to conventional risk factors. This raises the question which factors are perceived by the target group (patients with CVD) as priorities for further research.

**Methods:**

We carried out a survey to study priority setting for more research into conventional and sex- and gender-related risk factors according to 980 men and women with CVD or those at increased risk of CVD in the Netherlands. Data on conventional and sex- and gender-related risk factors were descriptively analysed, stratified by gender group.

**Results:**

The most frequently prioritised conventional factors according to men were heritability, overweight and unhealthy diet, while women most frequently listed stress, heritability and hypertension. The most frequently prioritised sex- and gender-related risk factors were depression or depressive feelings, migraine and having many caretaking responsibilities (men), and pregnancy complications, contraceptive pill use and early age at menopause (women). New research on sex- and gender-related risk factors was perceived roughly as relevant as that on conventional factors by men (mean 7.4 and 8.3 on a 1–10 scale, respectively) and women (8.2 and 8.6, respectively). Ethnic and gender minority groups placed more emphasis on risk factors related to sociocultural aspects (gender) than the majority group.

**Conclusion:**

Men and women with CVD or those at increased risk of CVD perceived new research on conventional and sex- and gender-related risk factors as a priority. These findings may guide researchers and funders in further prioritising new CVD research.

**Electronic supplementary material:**

The online version of this article (10.1007/s12471-020-01497-9) contains supplementary material, which is available to authorized users.

## What’s new

- In a survey, we asked patients with cardiovascular disease (CVD) or individuals at increased risk of CVD to prioritise various conventional and sex- and gender-related risk factors for further research.
- New research on sex- and gender-related risk factors was perceived roughly as relevant as that on conventional factors.
- Women and minority groups, in particular, assigned a high priority to sex- and gender-related risk factors.
- Ethnic and gender minority groups more frequently prioritised risk factors related to sociocultural aspects (gender) than the majority group.

## Introduction

Differences in the burden and risk of cardiovascular disease (CVD) between men and women have been widely reported across populations [1]. For instance, men develop CVD at a younger age and are more likely to present with coronary heart disease as a first sign of CVD, whereas women are more likely to develop heart failure or cerebrovascular disease as a first event [2]. The underlying mechanisms for this differential CVD risk are still largely unknown.

Differences in the occurrence and effects of conventional CVD risk factors may be partly responsible for the differential CVD risk [3]. For instance, women with diabetes mellitus have a 40% higher risk of incident coronary heart disease than men with diabetes [4]. In addition, emerging evidence shows strong associations between CVD and sex (a biological concept involving physical and physiological features) or sex-related factors. For instance, pregnancy-induced hypertension and preeclampsia are associated with future maternal CVD risk [5].

Moreover, novel evidence suggests that CVD risk might be associated with factors related to gender (a sociocultural concept including socially constructed roles, expectations, behaviours, expressions and identities of girls, women, boys, men and gender diverse people). For instance, recurrent acute coronary syndrome and major adverse cardiac events are associated with characteristics related to a more feminine gender (e.g. not being the primary earner or being primarily responsible for household work), independently of sex [6]. Since the mechanistic pathways remain unclear, there is a need for further investigation of sex- and gender-related risk factors in health research, as previously recommended, for example in the European Union’s research and innovation programme Horizon 2020 [7].

The increased attention for research into sex- and gender-related CVD risk in men and women raises the question which risk factors are perceived by the target group, for example men and women with CVD or those at increased risk of CVD, as priorities for further research and whether they perceive this additional research as relevant. The importance of patient and public involvement in health research has been increasingly acknowledged and implemented, for instance in the research agenda of the Dutch Heart Foundation [8]. Using patients’ everyday experiential knowledge in research has shown to improve the relevance, quality and applicability of research outcomes, as it complements professional and scientific knowledge [9, 10].

Therefore, we carried out a survey to study the prioritisation of conventional risk factors and of sex- and gender-related risk factors for future research according to men and women with CVD or those at increased CVD risk in the Netherlands.

## Methods

### Study design

We invited all 2369 members of a panel of the national Dutch CVD patients’ association (*Harteraad*) to take part in an online survey. This panel consists of CVD patients, individuals who are, in some way, related to CVD patients (partners, parents/legal guardians and caregivers) and individuals at increased risk of CVD (self-reported, e.g. those with diabetes, hypertension or overweight). The panel regularly receives surveys from healthcare professionals, researchers and policymakers on their experiences with cardiovascular health.

### Survey

On 15 November 2018, the panel received an email invitation with a hyperlink to an anonymous online survey (in Dutch) that was available for 7 weeks. Completion of the survey was voluntary. No personal reminder could be sent because the survey was anonymous and a general reminder would pose too great a burden on the panel at large. As the Dutch Medical Research Involving Human Subjects Act did not apply to this study, explicit ethical approval was not needed.

The survey included instructions and a brief introduction into the topic, followed by general questions on sociodemographic variables (age, country of birth, parents’ country of birth, sex, gender identity [personal perception of one’s own gender: man, woman, non-binary, gender fluid, transgender, transsexual, other], and educational level) and history of CVD. As a measure for priority setting, we asked respondents to choose a maximum of 3 out of 16 conventional risk factors (mentioned on the website of the Dutch Heart Foundation [11]; see Tab. 1 in Electronic Supplementary Material) on which they would like to spend a fictional research budget for new research within their gender group (‘Imagine that you can spend one million euros on more research on risk factors in your gender group. On which studies would you spend this money? You can choose a maximum of three’). Respondents could explain their choice in an open-text field (‘If you wish, you can elaborate on your choice of risk factor(s) below’).

As a measure of perceived relevance of new research on their chosen risk factors, we asked respondents to rate the importance of their budget allocation on a scale from 1 to 10 (’How important do you think it is to spend this money on new research on at least one of the three chosen risk factors? With ‘1’, you indicate that you think it is not important at all new research will be initiated, and with ‘10’, you indicate that you think it is very important new research will be initiated’).

We asked the same questions for 19 sex- and gender-related risk factors (Tab. 2 in Electronic Supplementary Material). These were characteristics, behaviours or conditions that were not considered conventional, such as hypertension and stress [11], and that were chosen based on previous research [3, 12–20].

Finally, to identify any other factors that might be related to CVD risk according to the respondents, they could suggest additional factors in an open-text field. All questions were closed-ended, except the 3 open-text field questions.

### Data and statistical analyses

The survey was anonymous and we did not track whether invitees filled in the form. Since it was not possible to check whether respondents who did not complete the survey filled in a complete second version at a later time, we used questionnaires with fully completed closed-ended questions in our data analyses (*n* = 980).

We analysed sociodemographic data and history of CVD descriptively, depicting them as percentage and mean ± standard deviation (SD). Gender group was based on the individual’s sex at birth combined with self-reported gender identity, and classified into cisgender men (those identifying as men or who were assigned the male sex at birth), cisgender women (those identifying as women or who were assigned the female sex at birth) and other gender groups. Ethnicity was defined using the validated country of birth indicator, by combining the individual’s country of birth with the parental countries of birth. We distinguished between a Dutch, non-Western migration and Western migration background [21, 22]. Educational level was classified into high level (higher vocational schooling or university) and other.

All data on conventional and sex- and gender-related risk factors were descriptively analysed as mean ± SD, stratified by gender group. All analyses were performed in IBM SPSS Statistics 25. The open-text field questions were summarised, but not further analysed.

### Post hoc sample

After the first survey, we distributed a shortened version of it to a convenience sample of 52 women with a non-Western migration background, who were recruited at local markets in Amsterdam, the Netherlands. We studied this group of women specifically because previous research has shown that they are a potentially overlooked high-risk group for CVD [23, 24]. Using this post hoc sample, we were also able to include a larger and more heterogeneous group of respondents with a non-Western migration background.

## Results

Of the 2369 panel members, 1198 commenced the survey, of which 980 completed it (41% response rate; 82% completion rate). A total number of 552 respondents (56%) were cisgender men (henceforth: men), 419 (43%) were cisgender women (henceforth: women) and 9 (1%) reported another gender identity (Tab. 1). The baseline characteristics of the gender groups were mostly similar, although more men and respondents reporting another gender identity were 60 years or older than women. Of the 218 uncompleted surveys, baseline characteristics of 144 respondents were available, which were similar to those of the 980 respondents who completed the survey (data not shown).

**Table 1:** Baseline characteristics, stratified by gender group

	Men (*n* = 552)	Women (*n* = 419)	Other gender (*n* = 9)
Age ≥60 years	85	49	89
Ethnic origin			
– Dutch	90	92	56
– Western migration background	9	7	44
– Non-Western migration background	1	1	0
High educational level	49	42	33
History of CVD	84	77	89
– <60 years at first event	62	79	75

All values are %

*CVD* cardiovascular disease

### Conventional risk factors

**Fig. 1:**
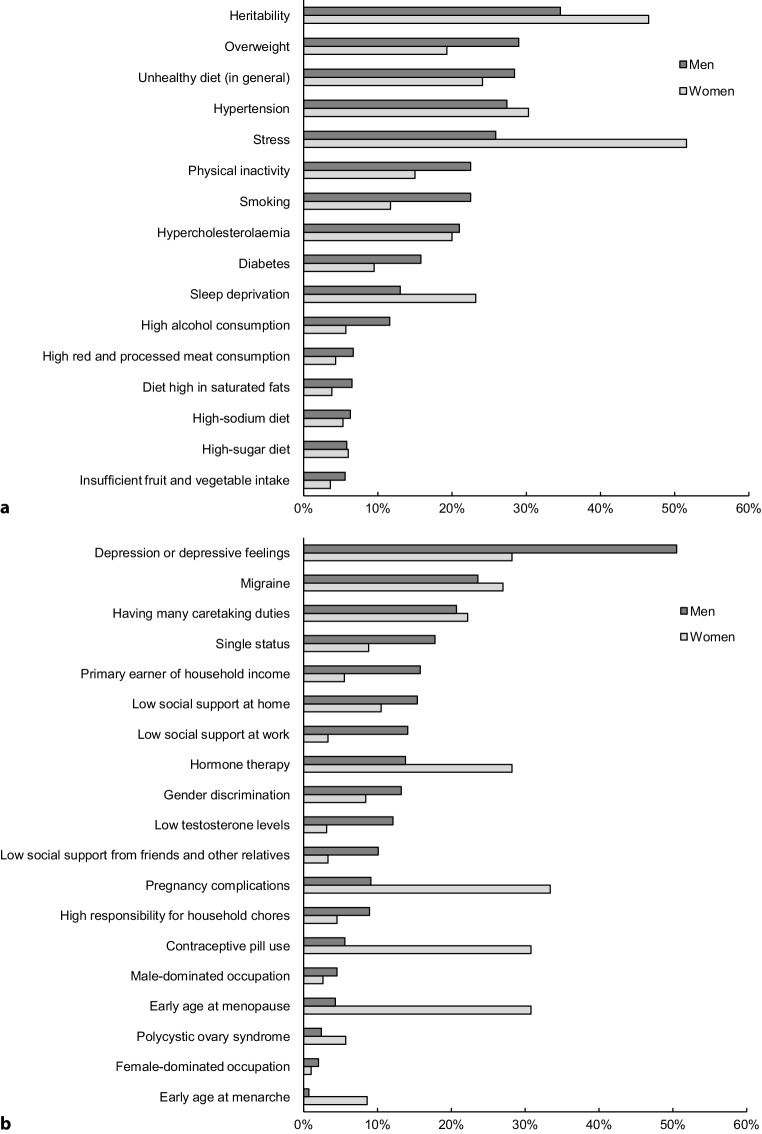
**a**, **b** Prioritisation of cardiovascular risk factors for further research (in percentages), by men and women, ordered from high to low (based on men). **a** Conventional risk factors. **b** Sex- and gender-related risk factors

Of the 16 conventional risk factors, heritability was the most frequently prioritised by men (35%), closely followed by overweight and a generally unhealthy diet (Fig. 1a). More than half of the women chose stress as a priority for further research, followed by heritability and hypertension. Around 26% of men and 30% of women elaborated on their choices (Tab. 3 in Electronic Supplementary Material). For instance, the high priority for heritability (frequently labelled as ‘genetics’) was often linked to a high CVD frequency in the family, without explicit consideration of the possibility of shared (unhealthy) lifestyles within the family.

### Sex- and gender-related risk factors

Of the 19 listed sex- and gender-related risk factors, more than half of the men chose depression or depressive feelings as a priority for further research, followed by migraine and having many caretaking responsibilities (Fig. 1b). Women most frequently prioritised pregnancy complications (33%), closely followed by contraceptive pill use and early age at menopause. Around 13% of men and 17% of women elaborated on their choices, with similar reasons as those for the conventional risk factors (Tab. 3 in Electronic Supplementary Material). For instance, some respondents reported that their pregnancy complication or migraine may have been the cause of their CVD.

### Budget allocation and additional risk factors

Among men, the mean score with regards to budget allocation for their 3 chosen sex- and gender-related risk factors was 7.4 ± 1.5, slightly lower than that for conventional risk factors (8.3 ± 1.2). Among women, the importance of sex- and gender-related risk factors was rated more similar to that of conventional risk factors (8.6 ± 1.2 vs 8.2 ± 1.4).

In the open-text field, around 18% of men and 25% of women mentioned additional risk factors, such as variations on previously mentioned risk factors (e.g. late age at menopause), comorbidities and associated treatments (e.g. cancer and chemotherapy), (chronic) medication use and environmental factors (e.g. particulate matter).

### Stratified groups

Among non-cisgender respondents (*n* = 9) and respondents with a non-Western migration background (*n* = 11), we observed slightly different response patterns. For instance, heritability was not a priority according to men and women with a non-Western migration background, whereas stress was additionally prioritised by men with a non-Western migration background. Furthermore, depression or depressive symptoms (by women with a non-Western migration background) and gender discrimination (by non-cisgender respondents) were additionally identified as priorities (data not shown).

### Post hoc sample

Results based on our post hoc sample of 52 women with a non-Western migration background were consistent with those based on the minority respondents of the panel. Hypertension, diabetes and stress (rather than heritability) were reported as priorities for further research. Moreover, the women in the post hoc sample more often deemed both sex-related (e.g. pregnancy complications) and gender-related risk factors (e.g. gender discrimination) priorities for new research than the majority panel did (data not shown).

## Discussion

Our study on patient perspectives on priorities for research shows that men and women with CVD or those at increased risk of CVD prioritise various conventional and sex- and gender-related risk factors for further research. New research on sex- and gender-related risk factors was perceived roughly as relevant as that on conventional factors. Women and minority groups, in particular, assigned a high priority to sex- and gender-related risk factors.

### Study limitations

Our study has some limitations. First, we targeted CVD patients specifically as we were interested in patient perspectives on prioritisation of risk factors for additional research. As a result, our findings may be less generalisable to the general Dutch population, which is younger and at lower CVD risk than our study population.

Second, the convenience sample of women with a non-Western migration background was recruited via an alternative route and most of these women did not have CVD. However, this group reported similar priorities as the ethnic minority subgroup included in the panel, suggesting that the lack of a CVD history does not explain the differences between the majority and minority groups.

Third, although our findings provide relevant information on priority setting for research in patients with CVD or in individuals at increased CVD risk, our study offers only limited insight into the reasons and motivations behind this priority setting. For instance, differences in reasons for prioritisation of risk factors between men and women could not be established due to a low response to questions requesting elaboration of choices. Nevertheless, our findings suggest that choices were frequently based on personal experiences. These experiences, for instance with sex- and gender-related risk factors in clinical practice, could be further investigated.

### Minority populations

Ethnic minority populations might be at a higher risk of CVD than majority populations [25, 26]. Accordingly, European guidelines have emphasised the need for more research on CVD risk in these groups [27]. We found that minority respondents prioritised risk factors related to sociocultural aspects (e.g. gender discrimination) frequently, more so than the majority panel. This may reflect a higher exposure to sex- and gender-related risk factors or an increased vulnerability to their impact among men and women from minority groups [28, 29].

## Conclusion

Men and women with CVD or those at increased risk of CVD perceived not only new research on conventional risk factors as relevant, but also research on sex- and gender-related risk factors. We recommend researchers to address both groups of risk factors in their future research to investigate unknown underlying mechanisms, as well as to improve existing or implement new awareness campaigns on already established risk factors. In addition, we encourage funders of CVD research to also consider sex- and gender-related risk factors by prioritising the further implementation of sex and gender in the design, analysis and interpretation of CVD research projects. Since risk differences between men and women have also been observed in other chronic diseases [30], we recommend considering priority setting for research into conventional and sex- and gender-related risk factors for other chronic diseases as well.

## Caption Electronic Supplementary Material

**Table 1.** Overview of conventional risk factors

**Table 2.** Overview of sex- and gender-related (SG) risk factors

**Table 3. **Main clusters of reported reasons for the choice of a maximum of three risk factors to spend the fictive budget of 1 million euros on
