# Supplementary material for: Patient perspectives on priorities for research on conventional and sex- and gender-related cardiovascular risk factors
Source: Neth Heart J. 2020 Oct 6;28(12):656–61. doi: 10.1007/s12471-020-01497-9 (PMC7683649; doi:10.1007/s12471-020-01497-9)
Supplement: Supplementary file 1 — Table 1. Overview of conventional risk factors [file 12471_2020_1497_MOESM1_ESM.docx]

**Table 1.** Overview of conventional risk factors

| **Conventional risk factors** |
| --- |
| Smoking |
| Being overweight |
| Drinking lots of alcohol |
| Little physical activity |
| An unhealthy diet (in general) |
| A diet high in saturated fats |
| A diet high in sugar |
| A diet high in salts |
| A diet with too much red or processed meats (for example, cold cuts, hamburgers, and sausages) |
| A diet with too little fruits and vegetables |
| Too little sleep/sleeping badly |
| Having high blood pressure (hypertension) |
| Having high cholesterol levels |
| Having diabetes |
| Heritability |
| Experiencing stress (for example, a stressful/emotional event such as death of a loved one, divorce, disease, moving) |
