# Supplementary material for: Patient perspectives on priorities for research on conventional and sex- and gender-related cardiovascular risk factors
Source: Neth Heart J. 2020 Oct 6;28(12):656–61. doi: 10.1007/s12471-020-01497-9 (PMC7683649; doi:10.1007/s12471-020-01497-9)
Supplement: Supplementary file 2 — Table 2. Overview of sex- and gender-related (SG) risk factors [file 12471_2020_1497_MOESM2_ESM.docx]

**Table 2.** Overview of sex- and gender-related (SG) risk factors

| **Sex- and gender-related risk factors** |
| --- |
| Early age at menopause |
| Early first period (menarche) |
| Taking the birth control pill |
| Complications during or just after pregnancy (for example, pre-eclampsia, high blood pressure during pregnancy, gestational diabetes, preterm baby/small for gestational age baby) |
| Having PCOS (polycystic ovary syndrome) |
| Having migraine |
| Treatment with hormones |
| Having low testosterone |
| Having a depression or depressive feelings |
| Having many care tasks (for example, being responsible for the care of children or parents) |
| Having high responsibility within the household for chores (such as cleaning, doing groceries, cooking) |
| Being the breadwinner of the household |
| Performing a male-dominated occupation |
| Performing a female-dominated occupation |
| Being single |
| Experiencing low support at home (for example, from partner/children/parents) |
| Experiencing low support from friends and other relatives |
| Experiencing low support at work |
| Unfair treatment, subordination or exclusion based on gender |
