# Supplementary material for: Patient perspectives on priorities for research on conventional and sex- and gender-related cardiovascular risk factors
Source: Neth Heart J. 2020 Oct 6;28(12):656–61. doi: 10.1007/s12471-020-01497-9 (PMC7683649; doi:10.1007/s12471-020-01497-9)
Supplement: Supplementary file 3 — Table 3. Main clusters of reported reasons for the choice of a maximum of three risk factors to spend the fictive budget of 1 million euros on [file 12471_2020_1497_MOESM3_ESM.docx]

**Table 3.** Main clusters of reported reasons for the choice of a maximum of three risk factors to spend the fictive budget of 1 million euros on

| **Reported reasons** |
| --- |
| The risk factor is relevant for the personal situation, e.g., it is most likely the cause of CVD or the person suffers from this risk factor. |
| The association between the risk factor and CVD is unclear to respondent and/or there is need for more scientific evidence (to convince themselves or medical doctors). |
| The risk factor is considered as more severe or more strongly related to CVD compared to other factors. |
| The risk factor is currently neglected or other risk factors are overstudied (there is enough scientific evidence). |
| Some risk factors were chosen because other risk factors were seen as own responsibility, choice, or fault. |

CVD, cardiovascular disease
